# Ticept: Wideband Electrical Properties Tomography by Tissue Composition Assessment With Quantitative HNaK Multinuclear MRI

**DOI:** 10.1002/mrm.70139

**Published:** 2025-10-24

**Authors:** Laura J. C. Barendsz, Kemal Sumser, Teresa Gerhalter, Rob M. C. Mestrom, Armin M. Nagel, Margarethus M. Paulides

**Affiliations:** ^1^ Department of Electrical Engineering Eindhoven University of Technology Eindhoven the Netherlands; ^2^ Institute of Radiology University Hospital Erlangen, Friedrich‐Alexander‐Universität Erlangen‐Nürnberg (FAU) Erlangen Germany; ^3^ Department of Neurology Medical University of Graz Graz Austria; ^4^ Medical Physics in Radiology German Cancer Research Center (DKFZ) Heidelberg Germany; ^5^ Department of Radiotherapy Erasmus University Medical Center Cancer Institute Rotterdam the Netherlands

**Keywords:** electrical conductivity, electrical properties tomography (EPT), mixture theory, permittivity, potassium MRI, sodium MRI, tissue water content

## Abstract

**Purpose:**

To develop a noninvasive method for estimating dielectric properties over a wide frequency range from 50 to 600 MHz. Existing methods are limited in accuracy and provide single‐frequency results. Furthermore, accurate knowledge of dielectric properties becomes more important, since medical device design and safety evaluation increasingly rely on computational modeling‐based evaluation.

**Methods:**

Tissue composition assessment was performed to find the most important factors for the dielectric properties, which were sodium, potassium, and water. These were measured with a 3D acquisition‐weighted density‐adapted stack‐of‐stars scheme (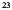
Na and 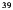
K) and an inversion recovery turbo spin echo sequence (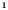
H). Measurements were conducted in muscle‐representative phantoms and six healthy volunteers. These measurements were used as input for mixture models, which were used to correlate the dielectric properties of the mixture with its constituents. Four different mixture models were tested to assess the feasibility of this method.

**Results:**

With the Maxwell–Garnett mixture model, a 7.9% error in the real relative permittivity and a 4.0% error in the effective conductivity are found for the phantoms. The in vivo values are in a similar range to those reported in literature.

**Conclusion:**

We presented the feasibility of estimating dielectric properties over a wide frequency band with quantitative MRI, leading to a new method: 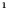
H
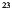
Na
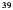
K‐TiCEPT. A discrepancy can be observed in the 50–200 MHz range, but the data aligns with literature from 200–600 MHz. These results show substantial intersubject differences in effective conductivity (29%), supporting the need for an accurate method to provide in vivo dielectric tissue properties.

## 1 Introduction

Over the last two decades, multi‐physics modeling has rapidly gained a pivotal position in virtual device design, model‐based evaluation, model‐based safety assessment, and real‐time treatment guidance [1, 2, 3, 4, 5]. Moreover, an increasing number of medical applications are employing electromagnetic (EM) fields across a broad spectrum of frequencies. Examples include MRI at different field strengths, hyperthermia cancer therapy, diathermy, microwave breast imaging, and in‐body communication [6, 7]. Virtual design and model‐based safety evaluation of such devices requires accurate knowledge of the tissue properties over a wide frequency range. This requires a more accurate knowledge of the tissue properties over a wide frequency range. In this study, the focus is on the in vivo dielectric properties of human tissue, that is, the real relative permittivity $\varepsilon_{r}^{\prime }$, and the effective conductivity $\sigma_{eff}$. These parameters describe the dielectric relaxation. For an ideal system, this can be described by the Debye equation [8], providing a fundamental explanation for how the behavior of polar molecules in an electric field can be modeled and interpreted. Building on this foundational understanding, the dielectric properties measured by Gabriel et al. [9] are de facto the gold standard for the dielectric properties of tissue in the range of 10 Hz‐20 GHz. These data are primarily based on ex vivo measurements, which do not account for physiological changes that affect tissue properties in vivo [10]. Furthermore, these data do not reflect the differences between populations [11, 12, 13]. These dependencies and differences between subjects point to the need for an accurate estimation of in vivo dielectric properties [14]. At the moment, no method can accurately assess the in vivo dielectric properties over a wide frequency range.

Magnetic resonance electrical properties tomography (MR‐EPT) enables noninvasive estimation of dielectric properties at the Larmor frequency. A review by Leijsen [15] shows that reconstruction methods include methods based on differential and integral equations. A recent development is the use of artificial intelligence (AI) to estimate the dielectric properties based on deep‐learning‐ or physics‐based approaches. All methods are associated with specific advantages and limitations. Approaches based on the differential equations are fast and easy to implement but suffer from inherent noise amplification. Integral methods are more noise robust but more challenging to implement and computationally expensive and often require knowledge of the incident electric and magnetic field strength that is inaccessible with MRI. AI‐based methods are fast and noise robust, but it is difficult to train the networks since there are no benchmark measurements [15, 16, 17]. These methods only work at the Larmor frequency. One study aiming for a wide frequency range was Liporace [18], who developed a method based on proton (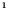
H) scans. Their theoretical study with literature values revealed errors within 16%, but ex vivo tests on muscle tissue revealed errors above 100% (effective conductivity) and 26% (real relative permittivity). Therefore, an accurate alternative method for determining dielectric properties in vivo over a wide frequency range is still urgently needed.

The existing methods suggest that water content alone is insufficient to estimate dielectric properties. Abasi et al. show that tissue properties in the radio frequency and microwave frequency range depend on the content of extra‐ and intracellular fluid [19]. Therefore, identifying the key contributors to the dielectric properties in these fluids and the rest of the tissue is crucial. Recent advances in multi‐nuclear MR have allowed for more precise quantitative measurements of these key components [20]. By combining the contributions of these different components, mixture models can be used to correlate the dielectric properties of the mixture with its constituents. We hypothesized that dielectric properties can be accurately estimated using mixture models based on the most relevant tissue constituents measurable with quantitative multinuclear MRI.

In this paper, we introduce a novel method called Tissue Composition EPT (TiCEPT) to determine the in vivo dielectric properties of human tissue across a wide frequency range from 50 to 600 MHz. This method assesses the most relevant tissue constituents using quantitative multinuclear MRI measurements. The research involves several key steps: first, identifying the primary contributors to the dielectric properties of human tissue; second, selecting the appropriate mixture model; third, obtaining MRI measurements to serve as inputs for the dielectric mixture models; fourth, validating the method by comparing the estimations with the gold standard probe measurements in phantoms; and finally, verifying the method in vivo by comparing the estimations with literature values reported by Gabriel et al. [9].

## 2 Theory

### 2.1 Tissue Composition

It is important to find the key elements in the tissue that influence the dielectric properties the most. Tissue in the human body consists of both water and non‐water components. In a typical 70‐kg male, approximately 60% of the body weight is water, while the remaining 40% comprises non‐water components. The water component can be further divided into intracellular fluid and extracellular fluid [21]. The non‐water components, on the other hand, consist of around 32% of proteins and fats [22].

As a result, water constitutes a large fraction of human tissue. Water consists of polar molecules that can align with the electric field, which increases the amount of polarization, and hence the real relative permittivity, because the real relative permittivity is a measure of electric polarizability [19]. Therefore, water is a major component determining the real relative permittivity in human tissue, as also shown by previous research [18, 23]. However, human tissue also consists of proteins and fats. Those components have lower real relative permittivity than water, which lowers global real relative permittivity. Hence, we deemed it crucial to include both water and fat with proteins in the real relative permittivity estimation.

The effective electrical conductivity of a material depends on the type and mobility of its charge carriers, which vary between water‐based and solid biological components. In water, conductivity is primarily due to the movement of dissolved ions that can freely migrate through the liquid. In contrast, in solid biological materials such as bone, fat, or connective tissue, conductivity is generally lower and arises mainly from limited ionic conduction through small amounts of interstitial fluid, as well as from polarization effects within the solid matrix. While water‐based systems allow all mobile ions to contribute to conductivity, in solid tissues the contribution is more restricted and depends on factors such as hydration level, porosity, and the presence of mobile ions within the tissue structure [21, 24].

Whether a current enters the intracellular fluid depends on the frequency of the current. This phenomenon can be attributed to the capacitive properties of the cell membrane consisting of a lipid bilayer [19]. At lower frequencies, the current travels around the cell in the extracellular fluid. However, at frequencies above ≈1 MHz, the current penetrates through the fatty cell wall, and therefore passes through the intracellular fluid as well. The most abundant cations in extracellular and intracellular fluid are [Na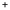] and [K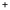], respectively. Consequently, at lower frequencies, sodium ions in the extracellular fluid are crucial for estimating dielectric properties, while at higher frequencies, potassium ions in the intracellular fluid also play a significant role [19]. Furthermore, [Na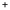] and [K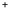] are at least 10 times more abundant in muscle than other cations [25]. For that reason, we assume that conductivity is primarily dominated by NaCl and KCl.

A MATLAB toolbox, based on Stogryn values [26], was used to determine the dielectric properties of NaCl given a specific salinity and temperature [27]. There are also different models available; however, these do not incorporate the salinity and temperature dependencies in the equations [28]. The temperature dependency is important in this research since the phantoms were at room temperature and the body temperature of the volunteer was assumed to be 35°C [29, 30]. This temperature difference leads to a difference of up to 25% difference in effective conductivity. Three different concentrations of NaCl and KCl solutions were measured to find the difference in effective conductivity between them. The resulting ratio (1.02) was used to scale the conductivity of KCl to NaCl, and in that way included in the mixture model.

### 2.2 Dielectric Mixture Models

The aim of this research is to retrieve the dielectric properties of human muscle with the highest accuracy possible over a large frequency band. Since individual components can be measured with quantitative magnetic resonance imaging, a model is needed to find the effective dielectric properties when these components are mixed. Mixture models account for the homogenization of a heterogeneous material, leading to macroscopic characterization with fewer parameters than needed for a complete description of the original object. One component is treated as the environment, also called the background (medium), and one (or more) component is (are) treated as the inclusion [31]. For biological tissue, we identified water with sodium and potassium as the background solution and fat and protein as the inclusion. The dielectric values of the background solution were determined with the MATLAB toolbox based on the Stogryn values as described in the previous section. Fat and protein were grouped and assigned the same dielectric properties. These properties were based on the measurements of peanut oil with a dielectric assessment kit (DAK‐TL2, Speag, Zurich, Switzerland). The real relative permittivity at 300 MHz was 2.4, and the effective conductivity was 0.003 S/m. The same values were used for the phantoms and in vivo estimation.

There is no clear consensus in the literature on which mixture model can be used best for biological tissue, as they all have certain assumptions and limitations. Hence, we compared four often‐used mixture models to find the mixture models with the lowest residual error. These will be described next.

#### 2.2.1 Maxwell–Garnett Equation

The Maxwell‐Garnett equation is the classical approach to determining dielectric properties from mixture models. The host medium is dominant, and inclusions are treated as isolated dipoles. The effective polarization arises primarily from the response of the inclusions embedded in the continuous host environment, making it a nonsymmetrical equation [31]. This leads to the equation [32]

(1) $$\varepsilon_{eff}=\varepsilon_{e}+3v\varepsilon_{e}\frac{\varepsilon_{i}-\varepsilon_{e}}{\varepsilon_{i}+2\varepsilon_{e}-v(\varepsilon_{i}-\varepsilon_{e})}$$

where $\varepsilon_{eff}$ is the effective permittivity of the mixture, $\varepsilon_{e}$ is the real relative permittivity of the background solution, $\varepsilon_{i}$ is the real relative permittivity of the inclusion, and v is the volume fraction of the inclusion. This equation assumes homogeneous dielectrics and inclusions that are spherical and small compared to the wavelength. It ignores the interaction between single inclusions and works mainly well for volume fractions below 0.1.

#### 2.2.2 Bruggeman Equation

The second equation is the Bruggeman equation which is a widely used symmetrical mixture model in which absolute equilibrium exists between the phases of the mixture. Polarization for the effective medium emerges both from the environment and from the inclusion [31]. This leads to the equation [33]

(2) $$(1-v)\frac{\varepsilon_{e}-\varepsilon_{eff}}{\varepsilon_{e}+2\varepsilon_{eff}}+v\frac{\varepsilon_{i}-\varepsilon_{eff}}{\varepsilon_{i}+2\varepsilon_{eff}}=0$$

with variables the same as in the Maxwell–Garnet equation. The Bruggeman equation also assumes the inclusions to be spherical, small compared to the wavelength, and furthermore assumes homogeneous dielectrics. The equation holds up to a volume fraction smaller than 0.8.

#### 2.2.3 Birchak and Looyenga Equations

The third and fourth equations can be represented by the power law models [31]

(3) $$\varepsilon_{eff}^{\beta }=v\varepsilon_{i}^{\beta }+(1-v)\varepsilon_{e}^{\beta }$$

The principle of these equations is that volume weights average a certain power of the real relative permittivity. The Birchak equation [34], with $\beta =\frac{1}{2}$, is based on the refractive index. The Looyenga equation [35], with $\beta =\frac{1}{3}$, is a more general derivation of the Bruggeman equation.

## 3 Methods

### 3.1 MRI Parameters

MRI measurements were performed using a Siemens 7T whole‐body MR system (Magnetom Terra.X, Siemens Healthcare GmbH, Erlangen, Germany). These measurements were used as inputs to the dielectric mixture models. Given that the only potassium coil with published human application results is designed for the calf and prior experience with quantitative imaging was available, we chose to study the calf muscle.

#### 3.1.1 Water Fraction Imaging

Imaging of the water fraction was used to determine the volume fraction of the inclusion in the dielectric mixture model [36]. An inversion recovery turbo spin echo sequence with the following parameters was used: TE = 14 ms, TR = 4160 ms, flip angle = 120°, FOV = 192×192 mm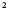, matrix size = 64×64, the number of averages = 1, TI = 200, 500, 750, 1000, 1250, 1500, 1750, 2000, 3000, 4000 ms, and acquisition time = 00:31 min:s [36]. A 28‐channel transmit and receive knee coil (Siemens Healthcare GmbH, Erlangen, Germany) was used for measurements. The T1 relaxometry data was analyzed using a MATLAB (R2023a, The MathWorks Inc., Natick, USA) script by Barral et al. [37]. The T1 times were correlated with the water fraction through a set of calibration phantoms [36]. The calibration phantoms consisted of water, sodium chloride, and sugar, which replicate various tissues in the human body with appropriate dielectric properties [38]. A reference curve was fitted using the T1 values (x‐axis) and the corresponding water fractions (y‐axis) of the calibration phantoms, which was subsequently used to estimate the water content of the muscle‐mimicking phantoms. The calibration curve can be found in the supplements.

#### 3.1.2 Sodium and Potassium Imaging

Sodium and potassium imaging were used to find the conductivity of the background solution used in the dielectric mixture model. The sodium and potassium images were acquired with a 3D acquisition‐weighted density‐adapted stack‐of‐stars scheme, with the following parameters for quantitative 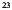
Na/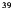
K: TR = 120/40 ms, TE = 0.3/0.4 ms, readout time = 10/5 ms, flip angle = 90°, rectangular excitation pulse of 500μs duration, nominal spatial resolution = 2.5×2.5×15/8×8×32 mm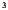, number of projections = 5384/2960, and acquisition time = 10:46/09:52 min:s. A dual‐tuned, circular polarized 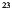
Na/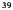
K calf coil with an inner diameter of 20 cm (Rapid Biomedical, Rimpar, Germany) was used for image acquisition. A 5‐compartment reference holder is integrated into the coil. The reference tubes for signal calibration were filled with different combinations of NaCl and K
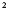
HPO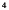 solution, leading to references with the following concentrations of [Na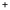] and [K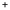]. [Na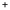]/[K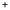] = 0.2/9.4, 0.5/8.2, 0.6/7.0, 0.7/5.9, 0.9/4.7 gr/L. The image acquisition and post‐processing pipeline was followed as described in [39]. This means that the 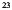
Na and 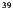
K images were corrected for partial volume effect, B0, relaxation, and distortion. The relaxation times found in [39] were used for the relaxation correction. The resulting concentrations of [Na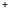] and [K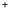] are reported as the mean concentration over 8 slices. An example of the calibration curves for the concentration of [Na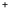] and [K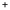] can be found in supplemental Figure S1.

### 3.2 Validation in Phantoms

Phantoms with EM, thermal, and magnetic resonance properties close to muscle tissue were used to test the method, allowing for precise validation, since the ingredients are known and benchmark measurements are possible. Phantoms consisting of demineralized water, agar‐agar (SaporePuro), peanut oil (supermarket: Arachide olie), protein powder (Body&Fit Egg White Protein Powder), and NaCl salt (supermarket: kitchen salt) show a strong similarity to the Gabriel measurements. Typical healthy tissue consists of 1.8% fat and 18.1% protein [40, 41] but, as mentioned before, there is a difference in dielectric properties between men and women, children and adults, and between healthy and unhealthy tissue.

In this investigation, the recipe shown in [42] was followed, except that part of the sodium was replaced by potassium (KCl > 99.5%, Labshop). The values used in the preparation in mass can be found in Table 1. 100 grams of protein powder contains 84 grams of protein, 3.1 grams of NaCl, and 12.9 grams of vitamins, minerals, and ash. Four phantoms with different compositions (indicated as volume fractions) were prepared, representing healthy (1.8% fat, 18.1% protein), obese (10% fat, 18.1% protein), dehydrated (1.8% fat, 25.5% protein), and a less protein muscle (1.8% fat, 12% protein). These muscle‐mimicking phantoms were placed in a larger container filled with 2.4 gr/L NaCl for the MRI measurements.

**TABLE 1:** Phantom ingredients in grams.

Constituent	“Healthy”	“Obese”	“Dehydrated”	“Less protein”
Demi water	1000	1000	1000	1000
Agar‐agar	16.8	16.8	16.8	16.8
Protein	92	103	142	57
Peanut oil	21	131	23	20
Potassium (KCl)	9.9	14.1	12.5	16.9

Benchmark dielectric property measurements were performed on the phantoms to measure the true dielectric properties. The measurements of dielectric properties were obtained with the Keysight P5004A Vector Network Analyzer (Keysight, Santa Rosa, California, United States) with a low‐loss coaxial cable used in combination with the Dielectric Assessment Kit for Thin Layers (DAK‐TL2) with the DAK12 probe (Speag, Zurich, Switzerland). This probe operates in a frequency range of 4 to 600 MHz and has a temperature range of 0°C to 50°C. The output power was set to 15 dBm. The calibration was performed by applying the open‐short‐load (OSL) calibration for liquid materials. The opening was measured by exposing the probe to air. The short was measured with copper tape. The load was measured by immersing the probe in a 5.84 g/L reference NaCl solution at room temperature. Once the calibration was completed, the calibration was validated by measuring the dielectric properties of a 9 g/L NaCl solution at room temperature. This method follows the guidelines published in [43]. To avoid measuring air bubbles, which is often the case in phantoms containing proteins, we shake the phantom until the measurements reach the highest stable measurement. This procedure was repeated ten times. The results were averaged and their standard deviation was calculated. After the measurements, the calibration validation liquid was measured again to check the system's calibration stability.

The results obtained with the dielectric mixture models were compared to the benchmark measurements to assess the accuracy of the method.

### 3.3 Verification in Healthy Volunteers

In vivo measurements were performed in healthy volunteers to verify whether the method was feasible in humans or not. The water fraction, the 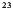
Na, and the 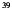
K images of the lower leg were acquired in 6 healthy volunteers (3 males and 3 females; age 32.6 ± 7.4 years; BMI 23.9 ± 3.4). The local ethical review board approved the study, and all volunteers provided informed consent prior to examination. The volunteers indicated that they did not suffer from any preexisting or acute illness, such as diabetes or hypertension.

The results obtained with the dielectric mixture models were compared with the literature values found by Gabriel et al. [9] to assess the feasibility of the method and to highlight the difference in dielectric properties between people.

## 4 Results

### 4.1 Validation in Phantoms

**FIGURE 1:**
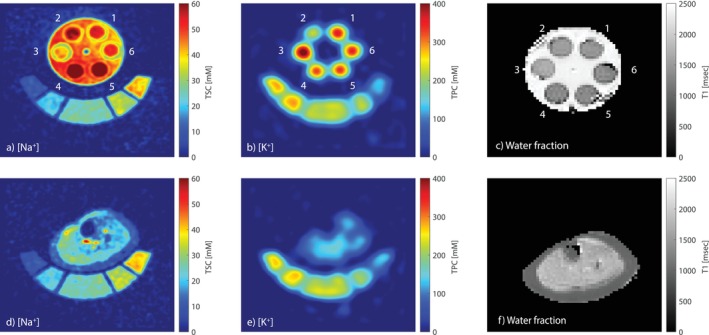
Uncorrected MRI images of the muscle‐mimicking phantoms (top row) and one volunteer (bottom row) for the [Na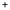], [K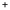], and water fraction measurements. The phantoms are 1. “healthy,” 2. “obese 1,” 3. “obese 2,” 4. “dehydrated 1,” 5. “dehydrated 2,” and 6. “less protein.” The reference phantoms for calibration of the [Na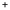] and [K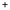] can also be seen in Figures (a–e).

The uncorrected sodium, potassium, and water fraction MRI images can be seen in Figure 1. The obese and dehydrated phantoms were placed twice in the setup to test reproducibility. The water fractions measured in the phantoms are: 77.7% (“healthy”), 74.8% (“obese 1”), 75.7% (“obese 2”), 71.6% (“dehydrated 1”), 72.7% (“dehydrated 2”), 80.3% (“less protein”). The water fraction for the volunteers was between 80.2% and 84.4%. The standard deviation of the T1 measurements was around 10%, which is comparable with the values in the literature [44, 45].

**FIGURE 2:**
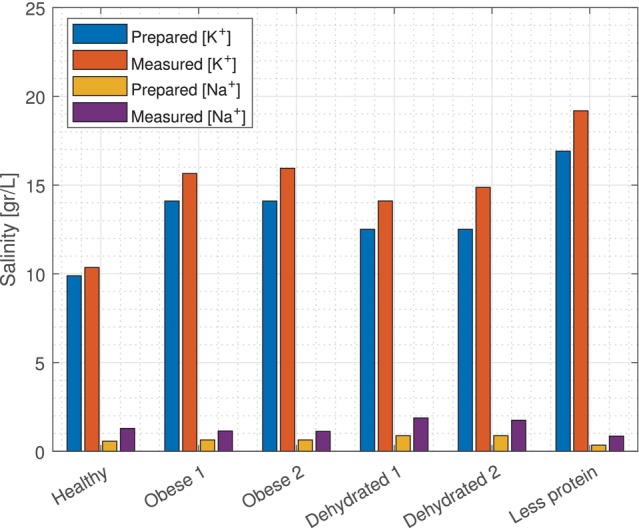
Salinity of [Na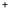] and [K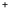] based on the preparation (ingredients) and the experiments.

Figure 2 shows the difference between the prepared and measured sodium and potassium values for the phantoms. This shows an overestimation with a mean difference between the prepared (ingredients) and experiment of 0.7 ± 0.2 g/L for [Na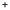] and 1.7 ± 0.7 g/L for [K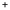]. This might be due to the assumption during relaxation correction that all six phantoms have the same relaxation times as used for in vivo muscle measurements. The exact measured values can be found in the supplements, where the uncorrected, relaxation‐corrected, and relaxation & partial volume corrected values are reported. Moreover, the resulting dielectric properties of the [Na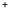] and [K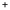] based on these measurements can also be found in supplemental Figure S2.

**FIGURE 3:**
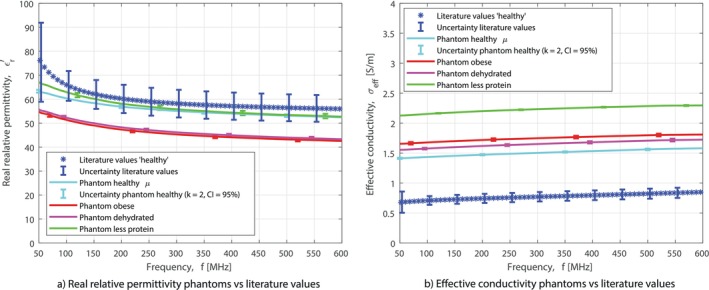
Measured dielectric properties of the phantoms against the parametric model based on measurement data by Gabriel et al. [9].

In Figure 3, the dielectric properties of the muscle‐mimicking phantoms are reported against the values from the research by Gabriel et al. [9]. The error bars indicate the uncertainty of the measurements of Gabriel et al. This variation is due to differences in physiological processes or other functional requirements. For the phantoms, the mean of ten measurements is shown with error bars that show the 95% confidence interval. It can be seen that the real relative permittivity and effective conductivity can be changed with different amounts of ingredients. The effective conductivity is higher than the physiological levels. Since the salt is incorporated into the protein powder, it cannot be reduced independently. We decided to keep the ratio between potassium and sodium comparable to physiological levels.

**FIGURE 4:**
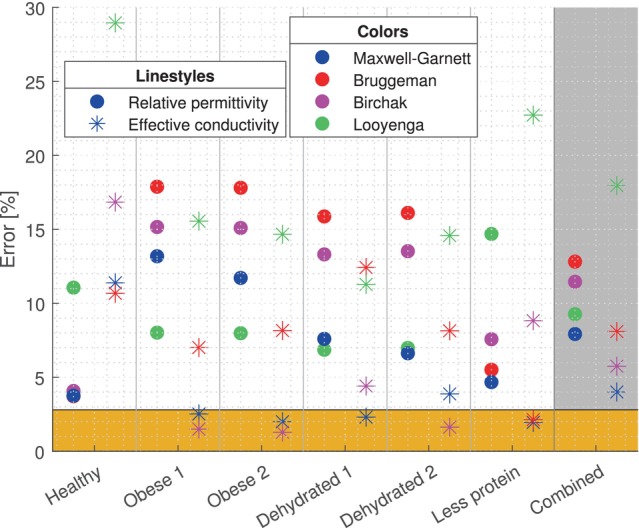
Relative error over the entire frequency band for the four mixture models compared, with the combined error of all phantoms in the last column. The orange bar shows the uncertainty of the DAK measurements of the muscle‐mimicking phantoms.

Figure 4 shows the error in real relative permittivity and effective conductivity for the estimation with the four different mixture models, compared to the DAK measurements of the muscle‐mimicking phantoms. From this figure it can be concluded that the Maxwell–Garnett equation works best for this application. For the Maxwell–Garnett mixture model, the specific errors at different Larmor frequencies can be found in Table 2 for the real relative permittivity and Table 3 for the effective conductivity. Taking the mean of the errors over the entire frequency band leads to a mean total error of 7.9% (real relative permittivity) and 4.0% (effective conductivity).

**TABLE 2:** Mean absolute percentage error for the real relative permittivity at specific frequencies and over the entire frequency range.

Phantom	50–600 MHz	65 MHz	130 MHz	300 MHz	400 MHz
“Healthy”	3.8%	12.9%	7.9%	1.4%	0.5%
“Obese 1”	13.2%	3.6%	3.8%	14.0%	17.1%
“Obese 2”	11.7%	5.1%	2.2%	12.2%	15.3%
“Dehydrated 1”	7.6%	9.9%	3.1%	6.4%	9.2%
“Dehydrated 2”	6.6%	11.4%	4.7%	4.6%	7.5%
“Less protein”	4.7%	16.0%	9.7%	1.2%	1.3%
Total	7.9%	9.8%	5.2%	6.6%	8.5%

**TABLE 3:** Mean absolute percentage error for the effective conductivity at specific frequencies and over the entire frequency range.

Phantom	50–600 MHz	65 MHz	130 MHz	300 MHz	400 MHz
“Healthy”	11.4%	7.2%	8.8%	11.6%	12.6%
“Obese 1”	2.5%	6.9%	5.0%	2.2%	1.2%
“Obese 2”	2.0%	6.3%	4.9%	1.7%	0.7%
“Dehydrated 1”	2.3%	7.1%	5.1%	1.8%	0.6%
“Dehydrated 2”	3.9%	1.0%	0.9%	4.0%	5.1%
“Less protein”	1.9%	5.8%	4.1%	1.6%	0.8%
Total	4.0%	5.7%	4.8%	3.8%	3.5%

### 4.2 Verification in Healthy Volunteers

The in vivo sodium, potassium, and water fraction MRI images can be seen in Figure 1. In vivo, we measured 0.4 ± 0.04/L for [Na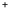] and 3.5 ± 0.4 gr/L for [K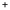]. The exact measured values, with and without correction, can be found in the supplements. These findings are in good agreement with previously reported results for similar MRI measurement [39], where they found a tissue concentration of 0.4 ± 0.02 g/L for [Na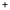] and 3.3 ± 0.2 gr/L for [K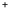]. The T1 time measured in volunteers was 1762 ± 50 ms, which led to a water fraction of 81% ± 0.9%. To the best of our knowledge, there are only two studies published about the T1 times of the calf muscle at 7T, which report a T1 time of 1552.5 ms [46] and 1864 ms [47]. The measured T1 time in this study lies between these values.

**FIGURE 5:**
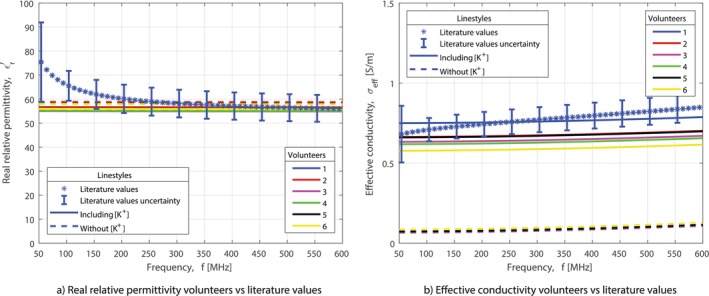
Estimated dielectric properties in vivo, with and without [K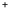], against the parametric model based on measurement data by Gabriel et al. [9].

Figure 5 shows the estimated real relative permittivity and effective conductivity, obtained with the Maxwell–Garnett equation, for the 6 volunteers, compared to the values in the literature found by Gabriel et al. [9]. Furthermore, it shows the estimation of the dielectric properties with and without [K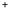] included. It can be seen that the impact of potassium on the real relative permittivity is negligible. However, the impact of potassium on the effective conductivity estimate is large, with an increase of at least a factor of 5 in the effective conductivity. This brings the dielectric properties of the volunteers, at least for a part of the frequency range, within the uncertainty range of the values in the literature.

A difference in the dielectric property values can be observed between the volunteers. The relative difference in effective conductivity between volunteer 1 and 6 is 29% (referenced to volunteer 6).

### 4.3 Robustness Analysis

First, the impact of the assumed body temperature on the dielectric property estimation was studied. When the body temperature was 1°C higher than assumed, this would lead to an error of 0.4%/°C for the real relative permittivity and 1.6%/°C for the effective permittivity.

Second, an error propagation analysis was performed to estimate the uncertainty associated with the measurements. The 10% error in the T1 measurements was incorporated, as well as the errors found between the prepared and measured sodium and potassium concentrations. When these values were used to estimate the uncertainties (*k* = 1), this gave an overall uncertainty of 5% for the real relative permittivity and 13% for the effective conductivity.

## 5 Discussion


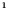
H
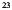
Na
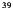
K‐TiCEPT shows an error of 7.9% for real relative permittivity and 4.0% for effective conductivity, which is substantially lower than the errors obtained in the only other reported wideband approach (≈16%). The errors are higher than the errors obtained by the supervised methods, which have errors around 5% [48, 49]. However, 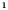
H
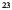
Na
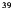
K‐TiCEPT has the advantage of a wide frequency band, also allowing comparisons to EPT at multiple field strengths, that is, frequencies. Furthermore, since it is based on the tissue composition, we expect that this method will aid our understanding of the foundations of dielectric properties in the human body.


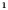
H
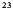
Na
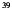
K‐TiCEPT exhibits a lower error for effective conductivity compared to the wEPT approach. This improvement is likely due to the inclusion of sodium and potassium measurements. Potassium significantly influences effective conductivity by at least a factor of 5, bringing the measured dielectric properties of the in vivo tissue close to the values reported in the literature, as illustrated in Figure 5.

An exact match between the estimated dielectric properties for volunteers and the data in the literature is not expected due to known population differences. This is reflected in the volunteer results, as shown in Figure 5, with the difference 29% between the volunteers. This emphasizes the need for a method to estimate the dielectric properties, since this is only the difference in dielectric properties between healthy volunteers. The difference between different populations can be expected to be greater, as cancer has been shown to change the water content and biochemistry of tissues, leading to different dielectric properties [13, 50]. Future studies should involve a larger cohort of volunteers and patients to map differences between populations, such as healthy and diseased individuals.

In the robustness analysis section, we discuss the impact of temperature. Although the uncertainty is not very large, the method would improve if exact in vivo temperature measurements were available. Especially when this method is extended to other tissues, this can be important. A good option for this temperature mapping would be multinuclear absolute magnetic resonance thermometry [51]. Furthermore, we show how the uncertainty is connected with the overestimation of the sodium and potassium measurements. However, the overestimation is expected to be lower in vivo, since the relaxation times in the correction are based on in vivo muscle measurements. This will also lead to lower errors.

In this study, we present an estimate of the dielectric properties of the entire calf muscle to show the feasibility of the method. However, if pathological alterations are present (e.g., muscle degeneration, edema, local injuries, or tumors), the dielectric properties will change locally. Therefore, it is important to move to a locoregional approximation of the dielectric properties or even a voxel‐wise estimation.

This method gives accurate results above 200 MHz. However, its accuracy in the 50–200 MHz range remains limited for the real relative permittivity estimation, as can be seen in Figure 5a. The discrepancy may arise from issues with the underlying mixture model. The current mixture model is not tailored for biological tissue, suggesting that the development of a tissue‐specific model could improve accuracy. These improvements could lead to even better results and might lead to an expansion of the possible frequency range.

In this study, we assumed that [Cl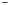] is the dominant anion influencing the dielectric properties of muscle tissue, simplifying the ionic balance to [Na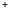] + [K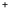] ≈ [Cl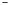]. However, [Cl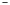] concentrations are lower than the combined [Na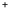] and [K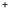] levels, indicating that other anions contribute to the conductivity [21]. Since the molarities in our study are relatively low, effective conductivity is mainly determined by the total ion concentration rather than ion‐ion interactions [52]. Given the range of molar ionic conductivities among different ions, we approximate the mean conductivity to be close to that of [Cl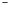]. This assumption is supported by the fact that our estimated conductivities align with values reported in the literature, although further in vivo validation is needed to confirm their accuracy under physiological conditions.

We note that the methods should be validated more extensively. This study shows the feasibility and accuracy of this tissue composition‐based wideband approach in phantoms and provides a first look at the estimation of in vivo dielectric properties. However, the in vivo estimation should be further validated, which is difficult due to a lack of a gold standard in vivo measurement method.

## 6 Conclusion

In this study, we explored the feasibility of using tissue composition to estimate wideband dielectric properties. Our findings demonstrate that the novel 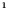
H
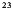
Na
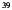
K‐TiCEPT method, which integrates quantitative and multinuclear MRI with the Maxwell–Garnett mixture model, effectively determines dielectric properties over a frequency range from 50 to 600 MHz. In phantoms, we achieved a mean error of 7.9% for real relative permittivity and 4.0% for effective conductivity. In vivo results showed real relative permittivity and effective conductivity values consistent with existing literature. However, from 50 to 200 MHz, the accuracy remains limited, most likely due to the assumed model; this should be improved in further studies. We observed considerable individual variations in effective conductivity (29%), which emphasizes the need for personalized in vivo dielectric property assessments. To enhance the utility of this method, further in vivo validation is required, for example, by comparison with B1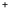 based assessment. Additionally, estimating dielectric properties in a larger cohort will provide deeper insights into interindividual differences and the impact of, for example, pathology, temperature, perfusion, and age.

## Supporting information




**Figure S1.** Calibration curves of [Na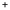], [K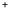], and the water fraction.
**Table S1.** Sodium and potassium concentrations for the uncorrected, relaxation‐corrected, and relaxation & partial volume corrected values for the phantoms as well as the volunteers.
**Figure S2.** Resulting real relative permittivity and effective conductivity of the [Na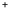] and [K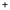] used in the mixture model.

## Data Availability

The data that support the findings of this study are available on request from the corresponding author. The data are not publicly available due to privacy or ethical restrictions.
