# Supplementary material for: Ticept: Wideband Electrical Properties Tomography by Tissue Composition Assessment With Quantitative HNaK Multinuclear MRI
Source: Magn Reson Med. 2025 Oct 24;95(3):1503–12. doi: 10.1002/mrm.70139 (PMC12746391; doi:10.1002/mrm.70139)

**Figure S1:** Calibration curves of [Na^+^], [K^+^], and the water fraction.


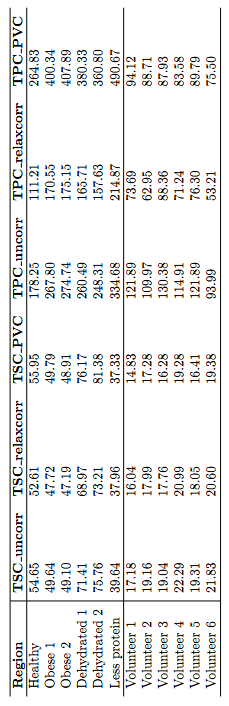

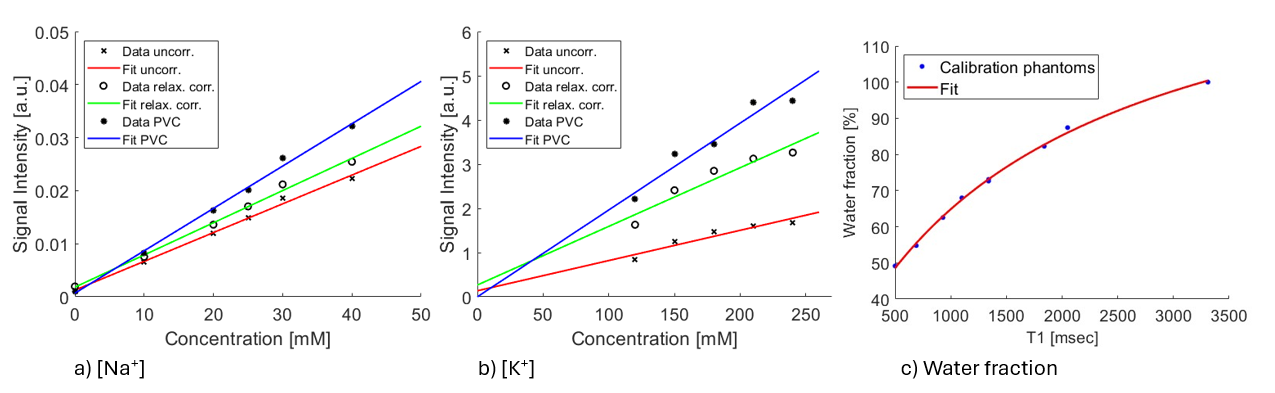


**Table S1**: Sodium and potassium concentrations for the uncorrected, relaxation-corrected, and relaxation & partial volume corrected values for the phantoms as well as the volunteers.

**Figure S2:** Resulting real relative permittivity and effective conductivity of the [Na^+^] and [K^+^] used in the mixture model.


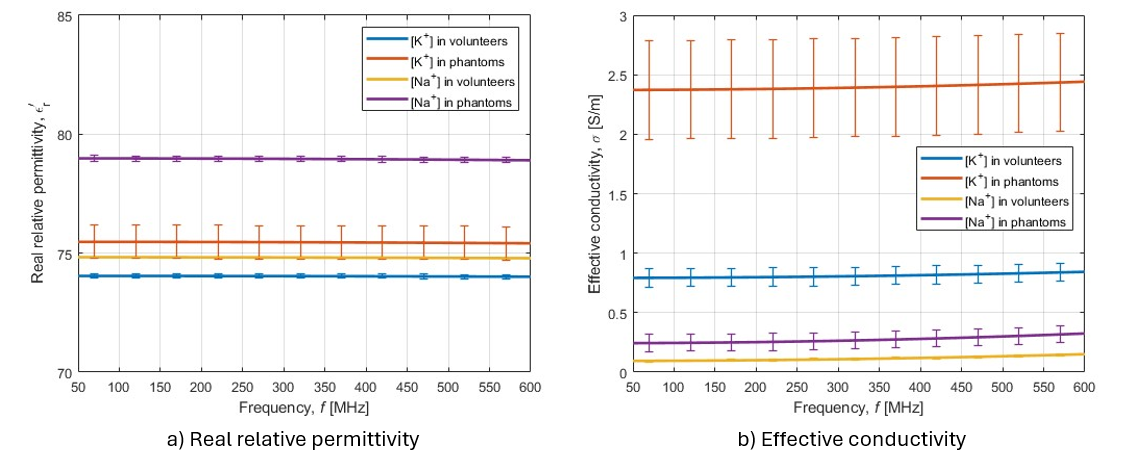

Supplement: Supplementary file 1 — Figure S1. Calibration curves of [Na], [K], and the water fraction. Table S1. Sodium and potassium concentrations for the uncorrected, relaxation‐corrected, and relaxation & partial volume corrected values for the phantoms as well as the volunteers. Figure S2. Resulting real relative permittivity and effective conductivity of the [Na] and [K] used in the mixture model. [file MRM-95-1503-s001.docx]
